# Sputtered Pd as Hydrogen Storage for a Chip-Integrated Microenergy System

**DOI:** 10.1155/2014/146126

**Published:** 2014-01-02

**Authors:** E. Slavcheva, G. Ganske, U. Schnakenberg

**Affiliations:** ^1^Institute of Materials in Electrical Engineering I, RWTH Aachen University, 52074 Aachen, Germany; ^2^Institute of Electrochemistry and Energy Systems, Bulgarian Academy of Sciences, 1113 Sofia, Bulgaria

## Abstract

The work presents a research on preparation and physical and electrochemical characterisation of dc magnetron sputtered Pd films envisaged for application as hydrogen storage in a chip-integrated hydrogen microenergy system. The influence of the changes in the sputtering pressure on the surface structure, morphology, and roughness was analysed by X-ray diffraction (XRD), scanning electron microscopy (SEM), and atomic force microscopy (AMF). The electrochemical activity towards hydrogen adsorption/desorption and formation of PdH were investigated in 0.5 M H_2_SO_4_ using the methods of cyclic voltammetry and galvanostatic polarisation. The changes in the electrical properties of the films as a function of the sputtering pressure and the level of hydrogenation were evaluated before and immediately after the electrochemical charging tests, using a four-probe technique. The research resulted in establishment of optimal sputter regime, ensuring fully reproducible Pd layers with highly developed surface, moderate porosity, and mechanical stability. Selected samples were integrated as hydrogen storage in a newly developed unitized microenergy system and tested in charging (water electrolysis) and discharging (fuel cell) operative mode at ambient conditions demonstrating a stable recycling performance.

## 1. Introduction

Nowadays the usage of hydrogen as a new environmentally clean energy carrier is already a reality. Numerous hydrogen fuel cells with various power outputs are already on the market and are being used for stationary, backup, and automotive applications. In the last years the boom in the portable electronic devices has led to an increased importance of the microfuel cells as an alternative power supply for consumer electronics such as multimedia, laptops, mobile phones, and so forth, as well as for powering of emergency and military equipment, different sensors, and medical devices.

The operating microfuel cells use three different fuel feeding systems: pure hydrocarbons (alcohols such as methanol and ethanol, formic acid, and ethylene glycol), pure hydrogen, and on-board generated hydrogen either from reformed hydrocarbons (like methanol) or from water. The interest in on-board hydrogen feeding is increasing gradually and these H_2_ *μ*FCs are more and more often considered as alternative of the Li-ion batteries. The advantages they promise include better efficiency, longer service life, lack of self-discharge, none or faster charging time. Moreover, H_2_ is nontoxic, nonirritant, and entirely environmentally friendly fuel with water being the only side product of its oxidation. Most of the hydrogen *μ*FCs reported in the literature work in a passive mode and consist of several membrane electrode assemblies (MEA) connected (most often horizontally) in a stack and a hydrogen reservoir [1, 2]. Preferably, H_2_ is stored in low plateau pressure metal hydrides to avoid pressure reduction components and is transported by out-of-storage diffusion. The O_2_ is transported to the reaction zone from the ambient air also by diffusion, for example, passively. For such “self-breathing” fuel cells with small lateral dimensions, the cooling by natural convection and radiation is sufficient and no active auxiliary parts are necessary. At the same time, because of the unforced reactants transport, the achievable power density is comparatively low (reported values vary in the range 2–300 *μ*W·cm^−2^) [3–6]. The low power output is compensated by the simplified design and the lack of energy consuming auxiliary (mechanical valves, feed pipes, and flow fields), so that all generated power is utilized by the consumer, requiring higher or lower power levels depending on the envisaged application. Reinecke et al. [7–9] presented a *μ*FC with integrated Pd hydrogen storage, electroplated on etched Si substrate, directly coupled to the anode and loaded with hydrogen from a gas phase or by an electrolytic process. The reported results showed that such *μ*FC at power output of 2 *μ*W·cm^−2^ withstands 170 h of continuous operation. The same authors later on have coupled the *μ*FC to an electrolyser cell, thus creating a fuel cell accumulator that acquires the ability to recharge the hydrogen storage on board. This rechargeable *μ*FC reached a power output about 300 *μ*W·cm^−2^, while the achieved discharge capacity was just 2.7% of the palladium theoretical hydrogen storage capacity. The design and performance characteristics of various microfuel cells stacks have been also reported [10–12].

Recently we have developed a bifunctional microenergy system with on-board H_2_ charging in which the same MEA is designed to work both in a fuel cell and in an electrolyser mode. The hydrogen storage material is an integrated part of the system. It is charged via water electrolysis and used to feed the system during its reverse operation in a fuel cell mode. This paper presents the results on the development, characterisation, and optimisation of the hydrogen storage layer. The material of choice is Pd, which is well known for its high hydrogen solubility and diffusivity, particularly in a thin film form, along with its excellent corrosion resistance.

## 2. Experimental

A series of palladium films were deposited on quartz glass substrates over a thin Ti-adhesion layer using Nordiko 2550 machine. The sputtering chamber was evacuated to a base pressure of 4 · 10^−9^ mbar using rotary vane and cryogenic pumps and then fed with argon gas at a constant flow rate of 100 sccm. The pressure was measured with a MKS Baratron pressure gauge and controlled by a throttle reducing the pumping speed of the cryogenic pump. The working distance between the metal target and the substrate and the dc power applied to Pd target were held constant (78 mm and 100 W, resp.). The pressure of the argon plasma (*p*_Ar_) was varied in the range of 5 · 10^−3^ to 1 · 10^−1^ mbar. For each sputter regime the deposition rate (nm/min) was determined. The deposition time was adjusted in accordance with the desired film thickness measured on a control sample via a liftoff process using surface profiler Tencor P-10. The sputtered Pd films were investigated systematically with reference to their crystallinity, surface morphology, roughness, and electrical conductivity. The crystal structure and phase identification were performed by X-ray diffraction (XRD) using an X-ray diffractometer Philips APD15 with CuK*α* (*λ* = 1.54178 Å) radiation over an angle range of 2*θ* = 10–90° with a step size of 0.02 2*θ* at constant rate of 0.02 2*θ*·s^−1^. The Rachinger method [13] was used to eliminate the *α*2 doublet of the CuK*α* radiation. The precise XRD peak position and peak broadening were obtained by fitting with the pseudo-Voigt function. Cell parameters were calculated using nonlinear regression analysis with “UNIITCELL” software [14]. The size of Pt crystallites was determined by Scherrer equation (1):

(1) $$\begin{array}{c} D=\frac{k\lambda }{\beta \cos\Theta }, \end{array}$$

where *D* is the average dimension of the crystallites, *k* is the Scherer constant in the range 0.85–1.0 in dependence on the crystal type (usually assumed to be *k* ≈ 1), **λ** is the X-ray wavelength, Θ is the Bragg angle and $\beta =\sqrt{\beta_{s}^{2}-\beta_{r}^{2}}$ is the peak broadening in radians, and *β*_*s*_ and *β*_*r*_ are the peak width at half-maximum intensity (FWHM) of the standard and the test samples, respectively.

The surface structure and morphology were studied by scanning electron microscopy (SEM) using ZEISS GEMINI 982 microscope with 3 kV acceleration voltage and magnification up to 200 000. The surface roughness was examined with Quesant Instrument microscope, model Q Scope 250. To evaluate the electrical properties of the sputtered layers the ohmic resistance was measured by a four-probe laboratory test station. The effect of the sputtering pressure on the electrochemically active surface and efficiency of the proceeding hydrogen adsorption/desorption phenomena were studied in 0.5 M H_2_SO_4_ aqueous solutions by cyclic voltammetry. To obtain information about PdH formation, galvanostatic polarization tests in the same solution were carried out. The changes in the electrical properties as a result of Pd hydrogenation were followed comparing the electrical resistance of the samples before and immediately after H_2_-charging.

## 3. Results and Discussion

The choice of dc magnetron sputtering for deposition of Pd as hydrogen storage in the developed chip-integrated microenergy system was justified by the compatibility of this method with the microsystem technology, its simplicity, and the possibility for easy control of the process parameters, which, in turn, allows tailoring of the surface structure, morphology, and thickness of the sputtered layer. The most important process parameters are the power of the electric field applied on the metal target, the working distance between the metal target and the substrate, and the pressure in the reactor. These factors determine the kinetic energy of the sputtered metal particles which is a key factor for the mechanism of the film growth and the properties of the deposited film. In a previous parametric study on dc magnetron sputtering of Pt it was shown that by optimisation of the sputter regime it is possible to modify essentially the film morphology, surface structure, and to some extent even the electrochemical performance [15]. In this research the applied power to the metal target (99.99% pure Pd) and the working distance to the substrate were kept constant, while the pressure of the argon plasma was varied in the range of 5 · 10^−3^–1 · 10^−1^. In view of the device compatibility, it was important to grow high-quality nanostructured Pd films on lattice nonmatching substrates. For this reason, the optimization of the process was performed on Pd films deposited on quartz glass. Table 1 summarizes all parameters of the selected sputter regimes.

As described in the experimental part, the thickness of the sputtered Pd layers was measured on a control sample structured via liftoff process and used to determine the sputter rate which, in turn, allows deposition of samples with defined thickness by simple adjustment of the process duration. The calculated values of the deposition rate at varying argon pressure are given in Table 2.

**Figure 1:**
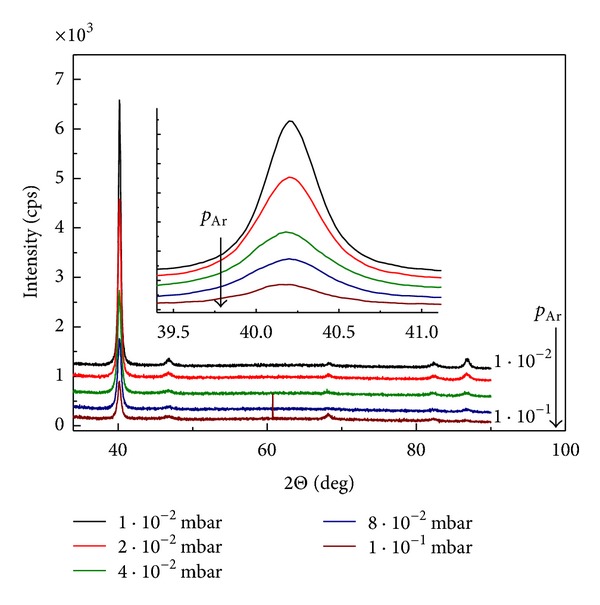
X-ray spectra of 120 nm thin Pd films deposited at various sputtering pressures.

In order to follow the effect of *p*_Ar_ in the sputtering chamber on the surface morphology and film structure, Pd samples were investigated using XRD and SEM analysis.  Figure 1 presents the obtained XRD spectra. It shows that the crystallography of Pd changes with the change in the pressure. At low pressure there is a clear orientation in the (111) crystallographic plane, while with the increase of *p*_Ar_ the intensity of the (111) peak decreases significantly and the film becomes more disordered. The size of the particles varies in the range 18–30 nm (Table 2) and decreases with the increasing *p*_Ar_.

**Figure 2:**
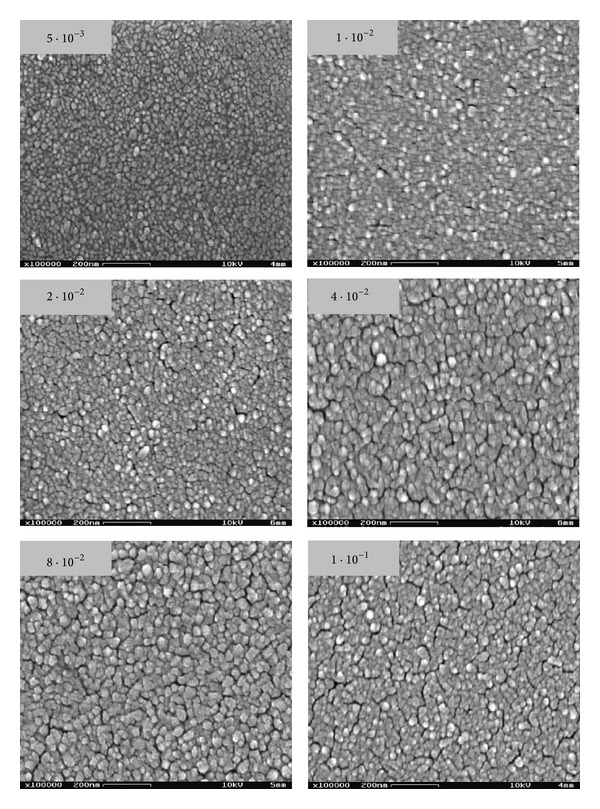
SEM images of as-deposited Pd layers at different sputtering pressures.

The SEM images in Figure 2 show that for all test samples the metal particles are homogeneously distributed forming a continuous compact film. The samples deposited at low pressure are smoother as with the increasing argon pressure a transformation to rougher and more porous surfaces is observed.

**Figure 3:**
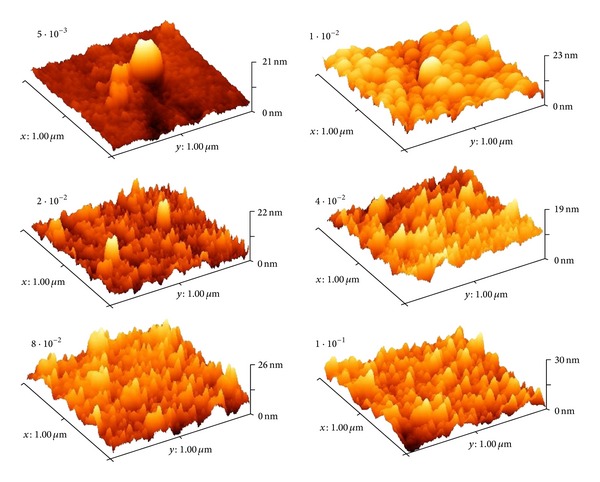
AFM images of Pd films deposited at various sputtering pressures.

**Figure 4:**
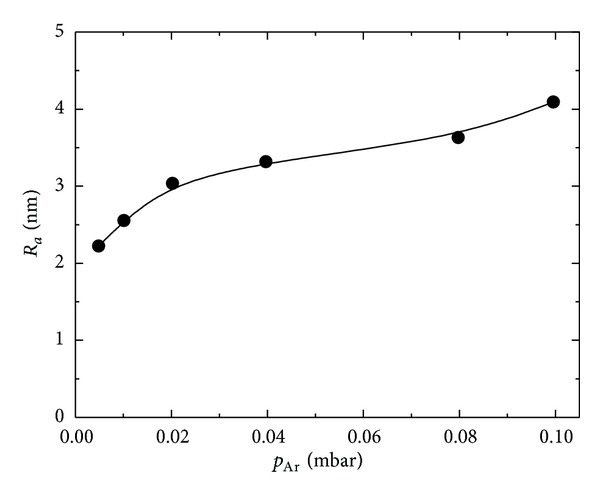
Influence of sputtering pressure on the surface roughness of the as-obtained Pd films.

The AFM images in Figure 3 are in accordance with the SEM analysis, confirming that Pd samples deposited at higher sputtering pressure have an increased surface roughness. The values of the roughness factor *R*_*a*_, calculated from the AFM data, are presented as function of the sputtering time in Figure 4.

The revealed effects are related to changes in the incident energy (*E*_inc_) of the sputtered Pd particles, representing the mean energy of the sputtered metal particle when it reaches the substrate [16]. When high-energy particles impact the substrate, they have long diffusion paths and are capable of rearranging on the surface forming a tightly packed layer. On the other hand, if the incident energy is low, meaning that most of the kinetic energy is lost during the transport, the diffusion over the substrate is negligible.

The incident energy *E*_inc_ is determined by the already mentioned three key variables (*P*, *WD*, and *p*_Ar_). Since in this study the only varying parameter is the sputtering pressure, it is entirely responsible for the changes in *E*_inc_ and thus, for the mechanism of growth and the properties of the sputtered films. At low pressure Pd particles have long mean free path between the collisions with the argon ions, resulting in a high incident energy and formation of a more compact and smooth metal films. In contrast, at higher *p*_Ar_, the sputtered metal particles collide more frequently with the argon ions, gradually losing their kinetic energy. The result is formation of sputtered layer with more open structure and larger surface area accessible for the electrolyte and the proceeding electrochemical reactions.

**Figure 5:**
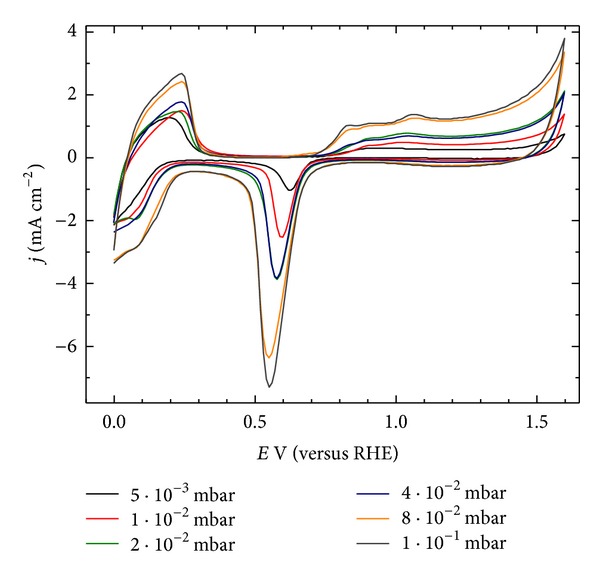
Cyclic voltammetry curves of the Pd layers deposited at varying sputtering pressures in 0.5 M H_2_SO_4_, potential scan rate 100 mV·s^−1^.

The effect of the changes in the morphology on the electrochemically active surface *S*_*A*_ and the proceeding hydrogen adsorption/desorption phenomena were investigated in 0.5 M H_2_SO_4_ aqueous solutions by cyclic voltammeter (Figure 5). The *S*_*A*_ values were calculated via integration of the area under the hydrogen adsorption peaks, according to a common electrochemical procedure using the charge of 210 *μ*C·cm^−2^ as a conversion factor [17]. The data presented in Table 3 show that the calculated electrochemically active surface SA and the roughness factor *f*, increase nonlinearly with the increase of the pressure, reaching a saturation at *p*_Ar_ = 8 · 10^−2^ mbar. They are in full accordance with the values of *R*_*a*_, determined by the AFM analysis.

The performed physical and electrochemical characterization of the sputtered test samples showed that the gradual increase of the argon pressure in the sputtering chamber leads to deposition of Pd films with less ordered structure, increased porosity, and more developed surface area effects that are favorable in view of the envisaged application of these films as hydrogen storage material. The values of *R*_*a*_ and *f* for the samples deposited at 8 · 10^−2^ and 1 · 10^−1^ are very close. Their cyclic voltammograms are similar as well, implying that both samples are appropriate for hydrogen storage, and presumably, should have similar storage capacity. On the other hand, due to the difference in their deposition rates (4.5 and 3.2 nm·min^−1^) the time required for the deposition of layers with equal thickness is longer in the case of higher pressure which is less attractive from the technological point of view. Based on all the results obtained, the process parameters *P*_Pd_ = 100 W/*WD* = 78 mm/*p*_Ar_ = 8 · 10^−2^ mbar were assumed as an optimal sputter regime.

**Figure 6:**
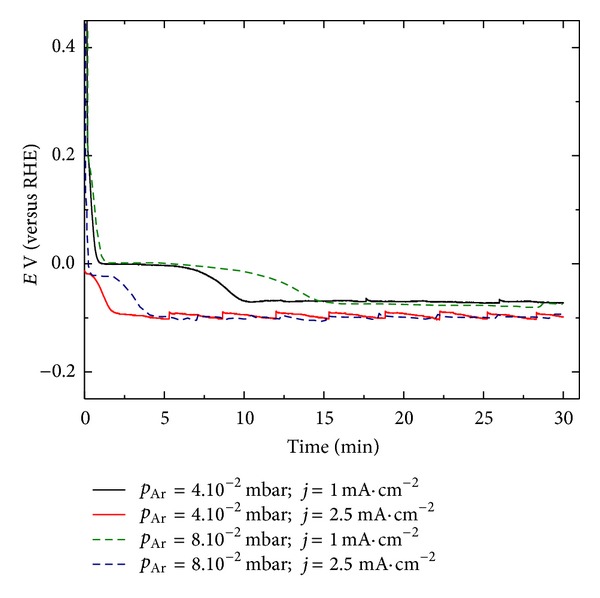
Electrochemical hydrogenation of 500 nm thick Pd layers deposited at sputtering pressure of 4 · 10^−2^ mbar (solid line) and 8 · 10^−2^ (dash line) at different charging currents.

It is known that the hydration of Pd goes through formation of two phases: PdH-a-phase that has a low Pd/H ratio (≤0.025) and hydrogen reach b-PdH with Pd/H ratio of about 0.6-0.7. In this study the sputtered Pd layers were charged via water electrolysis. The tests were carried out in galvanostatic mode at varying current densities. The charging curves presented in Figure 6 show two clearly distinguished potential steps which could be prescribed to the aforementioned formation of *α*- and *β*-PdH, respectively. The potential, at which these steps appear, shifts in negative direction with the increase of the applied charging current. It is seen that the hydrogenation process depends also on the morphology of the Pd film and is faster for the sample having more disordered porous structure.

**Figure 7:**
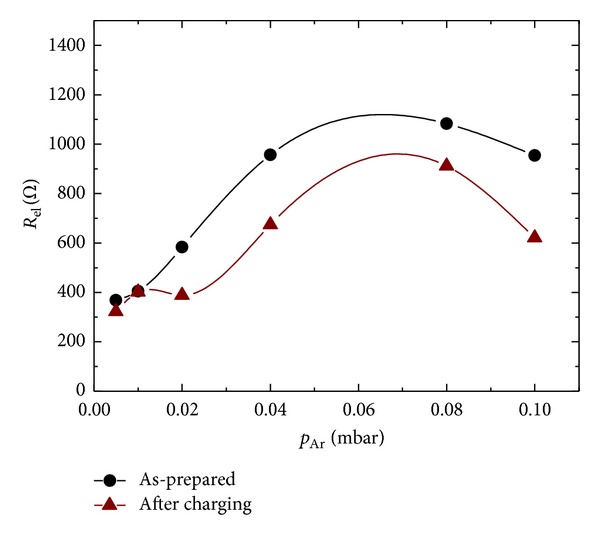
Electrical resistance of sputtered Pd films before and after hydrogenation.

In order to follow the changes in the electrical properties of the films as a function of the sputtering pressure and the level of hydrogenation, their electrical resistance was measured before and immediately after the electrochemical H_2_-charging tests, using the four-probe technique. The results presented in Figure 7 show a general trend to increase the resistance with the increasing pressure (up to *p*_Ar_ = 8 · 10^−2^ mbar) which is consistent with the data from the structural and morphological analysis. The lower the *p*_Ar_ is, the higher the incident energy of the sputtered particles is, leading to formation of smoother films with less defects and low porosity. These denser Pd layers have better electrical conductivity.

**Figure 8:**
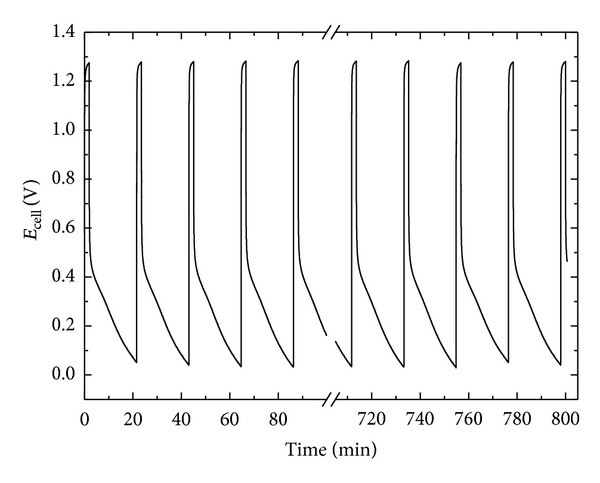
Performance tests of a microenergy systems with sputtered Pd as hydrogen storage; electrolysis/charge is carried out at *I*_ch_ = 5 mA, *t*_ch_ = 2 min; fuel cell/discharge is carried out at *I*_dch_ = 10 *μ*A, *t*_dch_ = 20 min.

Finally, a 500 nm thick Pd layer deposited at the established optimal sputter regime was integrated in a newly developed microenergy system and subjected to consecutive charging via water electrolysis, followed by a discharging in a fuel cell mode. The system demonstrated sustainable performance at ambient conditions for more than 100 reverse cycles (Figure 8). The design and fabrication procedure of the developed microenergy system as well as the detailed investigation of its electrochemical performance in both operative regimes will be reported in a following communication.

## 4. Conclusions

The research presented herein allowed establishing an optimal sputter regime (*P*_Pd_ = 100 W, *WD* = 78 mm, *p*_Ar_ = 8 · 10^−2^) for deposition of Pd layers with highly developed surface, moderate porosity, and long term mechanical stability which sustained in the whole range of film thicknesses tested (from 100 nm up to several *μ*m) and demonstrated a stable recycling performance as hydrogen storage in a chip-integrated unitized microenergy system.

## Figures and Tables

**Table 1:** Sputter parameters for the investigated Pd layers.

Target material	DC power (W)	Ar flow (sccm)	Pressure (mbar)	Target bias (V)
Ti*	250	55	4 · 10^−2^	225
Pd	100	55	5 · 10^−3^–1 · 10^−1^	337

*Adhesion sublayer.

**Table 2:** Dependence of deposition rate and crystallite size on the pressure.

*p*_Ar_ (mbar)	5 · 10^−3^	1 · 10^−2^	2 · 10^−2^	4 · 10^−2^	8 · 10^−2^	1 · 10^−1^
Sputter rate (nm·min^−1^)	7.8	7.5	7.34	6.9	4.5	3.2
Crystal size (nm)	33.0	27.5	22.0	18.0	17.5	19.0

**Table 3:** Electrochemically active surface (*S*_*A*_) and roughness factor (*f*) of Pd films deposited at various *p*_Ar_ calculated from the cyclic voltammeter curves.

*p*_Ar_ (mbar)	*S*_*A*_ (cm^2^)	*f*
5 · 10^−3^	11.5	5.8
1 · 10^−2^	20.3	10.2
2 · 10^−2^	27.2	13.7
4 · 10^−2^	27.6	13.9
8 · 10^−2^	46.5	23.4
1 · 10^−1^	48.9	24.6
